# Predicting Preterm Birth: An Evolving Landscape

**DOI:** 10.1111/1471-0528.18067

**Published:** 2025-01-13

**Authors:** Caroline Fox, Andrew Shennan

**Affiliations:** ^1^ Birmingham Women's and Children's NHS Foundation Trust Birmingham UK; ^2^ Guy's and St Thomas NHS Foundation Trust London UK

Recently, the international company Hologic has withdrawn fetal fibronectin (fFN), a commonly used biomarker for preterm birth prediction. This is related to quality controls and sourcing consumables. However, fFN is currently recommended by the National Institute for Health and Care Excellence (NICE) and the National Health Service in England and had been implemented widely across Europe and the United Kingdom. Therefore, withdrawal of fFN is forcing countries to reconsider their clinical approaches.

It is estimated worldwide that 13.4 million or 1 in 10 babies annually are preterm, before 37 completed weeks of gestation (WHO 2023). The earlier the birth, the higher the mortality and lifelong morbidity (D'Onofrio et al. *JAMA Psychiatry*. 2013;70:1231). Preterm birth is also a leading health cause of lost human capital (WHO 2023). Preterm birth rates are currently static and therefore initiatives to reduce prematurity and its complications are a priority.

Unfortunately, predicting and therefore preventing preterm birth is difficult as symptoms of threatened preterm birth can be vague and most do not go on to have their babies early. This led to consideration of tests to refine prediction of preterm birth and these include transvaginal cervical length (TCVL), as well as several cervico‐vaginal biomarker tests (fetal fibronectin [fFN], Actim Partus [phosphorylated insulin‐like growth factor binding protein‐1] and Partosure [placental alpha macroglobulin‐1]). Novel tests, such as cervical microRNA expression, cell‐free RNA, metabolomics and microbiome assessment, have yet to be widely evaluated.

NICE recommends transvaginal ultrasound measurement of cervical length and if unavailable or unacceptable fFN to define risk of preterm birth. A Cochrane systematic review (Berghella et al. 2019) reported that data are limited but knowledge of TVCL appears to prolong pregnancy by 4 days. This interval allows antenatal optimisation with corticosteroids and magnesium sulphate to improve survival and reduce morbidity. However, TVCL requires expertise and experience, and highly trained sonographers are not universally available at the point of care. fFN testing in contrast can be performed by speculum examination and was therefore the more widely adopted test. A low fFN, is reassuring that preterm birth within 7 or 14 days is unlikely with a negative predictive value of > 99% (Wing et al. *Obstet Gynaecol* 2017;130:1183). This allows women to be safely reassured and discharged. Cervical length alone has moderate prediction (area under curve to predict delivery < 30 weeks is 0.7), compared to fFN alone (0.89), but they can be used in combination for optimal prediction (Carter et al. *Ultrasound Obstet Gynecol*. 2019:55:357). Unfortunately, now fFN has been withdrawn, cervical scanning length is the only established alternative as other tests, such as Actim Partus, are not recommended by NICE. The Saving Babies Lives Care Bundle recommends TVCL in the midtrimester for high‐risk, asymptomatic, singletons and NICE similarly now recommends this for multiple pregnancies, facilitated by specialist clinics. Many clinicians are responsible for acute pregnancy care; however, particularly in the United Kingdom, clinicians are not trained in cervical length scanning. There is a need for TVCL to be available for symptomatic women as prediction is the key to allow targetted interventions and improved outcomes. To do this, we urgently need training programmes for TVCL while alternative biomarkers are evaluated to lead to enhanced prediction.

## Author Contributions

Both authors conceived and then wrote this article.

## Conflicts of Interest

The authors declare no conflicts of interest.

## Data Availability

Data sharing is not applicable to this article as no new data were created or analyzed in this study.

